# Keeping it in the family: Malignant Hyperthermia - how we predict, recognise and treat it

**DOI:** 10.1016/j.bjane.2025.844645

**Published:** 2025-05-28

**Authors:** Jonathan G. Bilmen, Pawan K. Gupta

**Affiliations:** United Kingdom Malignant Hyperthermia Unit, St. James’ University Hospital, Leeds, UK

Malignant Hyperthermia (MH) is a rare but potentially fatal complication of general anaesthesia. First described by Michael Denborough in 1960,^1^ the reaction occurs when volatile anaesthetic agents trigger uncontrolled calcium release from skeletal muscle fibres, resulting in massive consumption of energy in the form of Adenosine Triphosphate (ATP) hydrolysis. This uncontrolled calcium release into the muscle cell sarcoplasm is primarily through the ryanodine receptor type 1, which leads to activation of the myosin-actin complex (the muscle machinery) and hence increased muscle tone. Furthermore, the calcium reuptake into the muscle is an active process, requiring even more energy being used.^2^ This vicious cycle continues until either the cell dies, or the ryanodine receptor is switched into the ‘off state’, which with our current pharmacology, is achieved by use of dantrolene.

By understanding these basic biochemical and physiological principles, the symptoms that patients experience with MH crises become self-evident and easier to understand.

An increase in muscle work leads to an increase in energy consumption and heat production. There is an increase in metabolism in the muscle cell to meet the energy requirements, leading to both aerobic (oxygen derived) and anaerobic respiration to produce ATP. The aerobic respiration leads to an increased oxygen requirement and an increased production of carbon dioxide, whereas the anaerobic respiration leads to lactic acid production, which is then removed by the circulation.

Eventually, the muscle cells become exhausted and may leak or even necrose, either from overstimulation or through hyperthermia. Once the cell leaks or dies, it releases its contents into the surrounding tissues, which may include myoglobin and/or potassium, amongst many other compounds (known as rhabdomyolysis).^2^

The clinical signs and symptoms of MH arise from the above underlying pathophysiological mechanisms. Both the Clinical Grading Scale^3^ and the Leeds Clinical Category Scale^4^ highlight the considerable variation in how MH manifests, which complicates definitive diagnosis. The likelihood of MH can vary widely, from 0.96 (near-certain) to as low as 0.07 (unlikely), depending on the specific clinical features observed^4^ variable clinical presentation and in test positivity rates, as also seen in Brazilian clinical data^5^ remain key diagnostic challenges based on clinical presentation alone.

The treatment for MH is a 3-pronged response:^6^

• Remove the triggering agent;
• Give dantrolene;
• Treat the symptoms and signs as they present.

It should be clarified that MH can be triggered by a variety of volatile anaesthetic agents. It is not limited to just halothane or sevoflurane. Indeed, it has been documented that ether, chloroform and methoxyflurane (currently used as an inhalational analgesic agent is some countries under the brand of Penthrox®) can trigger an MH reaction.^7^^,^^8^ Furthermore, some can trigger after multiple exposures to volatile agents. In the UK, some patients have had approximately six general anaesthetics before presenting with a suspected MH reaction *(personal observations of the authors)*. The removal of halothane from the anaesthetic armamentarium doesn’t remove the risk of MH from a patient when exposed to an inhalational anaesthetic. In fact, the only inhalational agents that can be safely used in an MH susceptible patient are nitrous oxide^9^ or xenon.^10^

The current known aetiology of MH lies in the expression of three intracellular proteins (and their variants) that are core to the mechanism of calcium release in skeletal muscle:^11^

• The ryanodine receptor type 1 (RyR1);
• The voltage sensitive calcium channel, Cav_1.1_ (previously known as the dihydropyridine receptor);
• The protein STAC3 – a molecule involved in the linkage between Cav_1.1_ and RyR1.

Whilst Cav_1.1_ allows Calcium to enter the cell from the surrounding tissues when activated by a depolarising sarcolemma, its primary mechanism of triggering muscle contraction is through a direct linkage between Cav_1.1_ and RyR1 within the cell. Under normal conditions, an activated Cav_1.1_ stimulates RyR1 to open and release calcium from intracellular stores, which in turn leads to muscle contraction (known as excitation-contraction-coupling). It is therefore clear that any disruption to this pathway can lead to changes in the control of calcium release.^2^

Family history and genetics are key to understanding the cause of MH susceptibility. Until recently, it was believed that MH was an autosomal dominant condition, that is, with one parent being MH susceptible, the offspring had a 50/50 chance of also being susceptible.^12^ Indeed, in most cases, MH susceptibility occurs when the causative gene is inherited from one parent. However, our research and understanding has now evolved to demonstrate that the aetiology of MH may not be as linear or ‘Mendelian’ as was once thought.^11^ Whilst there are some genetic variants in one of the above-mentioned proteins that will almost certainly lead to MH susceptibility, there are others which may or may not be pathogenic, that is, the cause of a potential MH presentation. This is important to remember when assessing a patient and their family history for risk of MH.

In a letter to the BJAN published in this issue,^13^ Silva and her colleagues are highlighting a concern that the Brazilian Malignant Hyperthermia diagnostic centres have reported receiving multiple requests from healthcare professionals for lists of surnames associated with MH-positive patients to assess individual MH risk. Brazilian naming conventions add further complexity to this issue.^14^ Unlike many cultures, Brazilian surnames follow a distinct pattern influenced by Portuguese traditions, where a child typically may receive a given first name, mother’s maiden name and the father’s last name. The footballer Neymar Jr.’s full name (*Neymar da Silva Santos Júnior*) reflects this structure "Neymar" (given name), "da Silva" (meaning "of the forest") and "Santos Júnior" (from father’s surname).^4^ Surname ancestry data in Brazil shows a diverse distribution, with Italian, German, Iberian, Eastern European, and Japanese influences being most common.^15^ Given this variability and the fact that surnames may not reliably indicate paternal or maternal lineage any assumptions about MH susceptibility based solely on surnames are scientifically unfounded. In addition, it will label a large population at risk of MH. As mentioned above, susceptibility can also arise through *de novo* mutations and compound heterozygosity. To assume that a surname is suggestive of increased risk of MH is inappropriate. There is no substitute to assessing a patient’s *a priori* risk for MH other than by taking a detailed clinical and family history, should the concern arise. Thus, while familial history remains an important consideration, MH risk assessment must rely on clinical evaluation, genetic testing and *In Vitro Contracture Test* (IVCT) rather than surname-based presumptions.

In the UK, the incidence of a true MH reaction is approximately 1 in 115,000 anaesthetics (*Prof. Hopkins personal communication*, 2025)*.* This illustrates how rare it is to see a patient with MH. It is very likely that most anaesthetists will never see an MH reaction during their entire career. Therefore, an anaesthetic practitioner cannot rely on clinical experience alone to train/learn on how to deal with such an incident when it occurs. Learning to treat these patients must be augmented with supplemental interventions – such as reading and simulation-based training.^16^ Simulation-based training allows clinicians to learn and develop knowledge and skills in a safe environment as well as practice those skills in advance of being confronted with ‘the real thing’. Those skills however are not limited to just practical skills but also developing team-working and leadership attributes as well. Some scenarios are highly effective in demonstrating a clinician’s bandwidth, that is, the ability to deal with multiple concerns and challenges at a single point in time. When a clinician’s bandwidth is exceeded, that is where potential errors and mistakes occur, with heightened stress experienced in the performing clinician, potentially becoming overwhelmed. Simulation-based training helps to address these areas and improve the ability of the healthcare professional to cope with the clinical situation when presented to them. It has been employed in many courses worldwide and is a cornerstone of clinical training. It has been demonstrated to be highly effective in the Advanced Life Support (ALS) course^17^ whilst the Anaesthesia, Trauma and Critical Care (ATACC) course uses a mixture of simulation-based training and immersive experiences to educate their students. The aim is that by the conclusion of such interactive courses, the practitioner should have developed a ‘muscle memory’ in dealing with similar situations when presented in real life.

In Neville’s et al. article in this issue of BJAN, the researchers demonstrate the benefit of simulation-based training in addressing an MH crisis.^18^ All 30 anaesthesiology residents that were enrolled into the research study received classroom teaching on how to treat a patient experiencing an MH crisis one week prior to a simulation-based assessment. This was a critical consideration, as designing an MH simulation scenario presents significant challenges due to variable clinical manifestations.^4^ The candidates were paired up and split into three groups. One group (5 pairs) had no access to a cognitive aid, one group had access to a poster as a cognitive aid and one group had access to a mobile phone based cognitive aid. All three groups treated the patient in the simulation-based scenario effectively, with successful outcomes achieved and the main treatment requirements delivered. However, those groups with a cognitive aid (poster or mobile phone based) demonstrated higher scores when assessed for their technical and non-technical skills. This article illustrates the importance of both simulation-based training and assessment, coupled with the use of a cognitive aid being immediately available should such an incident arise.

The use of cognitive aids in the UK are variable. The authors of this article work in multiple institutions and note that the visibility of the UK MH guidelines in case of crisis is varied. Some centres display the guideline as a poster on the wall, others keep them attached to the side of the anaesthetic workstation and some place them in a folder either alongside the dantrolene or in theatre. Given the results of the above study, it would seem reasonable to conclude that all centres where MH could be a potential sequela of anaesthetic intervention should have guidelines immediately available and probably immediately visible. Anything that can be done to improve the effectiveness of treatment should be actively encouraged and this trial demonstrates that cognitive aids may play a significant role in improving our ability to treat patients effectively. It should be noted that there was no evidence to suggest a reduction in time to successful treatment of these patients in the simulation. It would be interesting to know whether this is simply a reflection of the small sample size or whether this is a true reflection that there is little that a cognitive aid can offer in delivering more timely treatment. For example, the older formulations of dantrolene can take a significant amount of time to mix, which leads to delays in administration and effective treatment in an episode of MH. The presence of a cognitive aid will not change this mixing time, though the introduction of newer formulations of dantrolene (for example, Ryanodex® or Agilus®) may have a significant impact in terminating an MH reaction sooner.

In summary, MH is a disease which can strike with little warning. Regardless of family history, we as anaesthetic practitioners should be aware that it can present at any time. The only way to conclusively rule out a patient from being susceptible to MH is through IVCT with a negative result. Simulation-based training and cognitive aids are important to ensure that, when a clinician needs to deal with a patient experiencing an MH crisis, they can deliver the safest and optimal care possible.

## Conflicts of interest

The authors declare no conflicts of interest.
